# New Insights Into the Regulation of Natural-Killer Group 2 Member D (NKG2D) and NKG2D-Ligands: Endoplasmic Reticulum Stress and CEA-Related Cell Adhesion Molecule 1

**DOI:** 10.3389/fimmu.2018.01324

**Published:** 2018-06-18

**Authors:** Shuhei Hosomi, Joep Grootjans, Yu-Hwa Huang, Arthur Kaser, Richard S. Blumberg

**Affiliations:** ^1^Division of Gastroenterology, Department of Medicine, Brigham and Women’s Hospital, Harvard Medical School, Boston, MA, United States; ^2^Department of Gastroenterology, Osaka City University Graduate School of Medicine, Osaka, Japan; ^3^Department of Gastroenterology and Hepatology, Academic Medical Center, University of Amsterdam, Amsterdam, Netherlands; ^4^Division of Gastroenterology and Hepatology, Department of Medicine, University of Cambridge, Addenbrooke’s Hospital, Cambridge, United Kingdom

**Keywords:** natural-killer group 2 member D, natural-killer group 2 member D-ligand, murine UL16-binding protein like transcript 1, UL16 binding protein 1, endoplasmic reticulum stress, CEA-related cell adhesion molecule 1

## Abstract

Natural-killer group 2 member D (NKG2D) is a well-characterized activating receptor expressed by natural killer (NK) cells, NKT cells, activated CD8^+^ T cells, subsets of γδ^+^ T cells, and innate-like T cells. NKG2D recognizes multiple ligands (NKG2D-ligands) to mount an innate immune response against stressed, transformed, or infected cells. NKG2D-ligand surface expression is tightly restricted on healthy cells through transcriptional and post-transcriptional mechanisms, while transformed or infected cells express the ligands as a danger signal. Recent studies have revealed that unfolded protein response pathways during endoplasmic reticulum (ER) stress result in upregulation of ULBP-related protein *via* the protein kinase RNA-like ER kinase-activating factor 4-C/EBP homologous protein (PERK-ATF4-CHOP) pathway, which can be linked to the pathogenesis of autoimmune diseases. Transformed cells, however, possess mechanisms to escape NKG2D-mediated immune surveillance, such as upregulation of carcinoembryonic antigen (CEA)-related cell adhesion molecule 1 (CEACAM1), a negative regulator of NKG2D-ligands. In this review, we discuss mechanisms of NKG2D-ligand regulation, with a focus on newly discovered mechanisms that promote NKG2D-ligand expression on epithelial cells, including ER stress, and mechanisms that suppress NKG2D-ligand-mediated killing of cancer cells, namely by co-expression of CEACAM1.

## Introduction

Natural killer (NK) cells were originally identified as lymphocytes with cytotoxic reactivity against several types of cancer cells (1, 2), and are considered to be part of the innate immune cell compartment/family/pool because of lack of rearranged antigen-specific receptors by somatic recombination. Both activating and inhibitory receptors can regulate NK cells activity and recognize target cells (3). Inhibitory receptors, including killer cell immunoglobulin (Ig)-like receptors (KIRs) and leukocyte Ig-like receptors in humans, Ly49s in mouse, and CD94-Natural-killer group 2 member A receptors in human and mouse, recognize major histocompatibility complex (MHC) class I. If NK cells encounter cells that express MHC class I, an immune response against these cells is prevented by the inhibitory signals through receptor–ligand interactions. In contrast, cells in which MHC class I is downregulated, for example, in virus-infected cells or cancer cells, NK cells are activated by the lack of inhibitory signals, which makes the “diseased” cells prone to NK cell-mediated killing (the missing-self hypothesis) (4). The inhibitory function is mediated through immunoreceptor tyrosine-based inhibitory motif (ITIM) in the cytoplasmic domain of these inhibitory receptors (5).

Besides inhibitory receptors, numerous activating NK receptors, such as CD94-Natural-killer group 2 member C, natural-killer group 2 member D (NKG2D), NKp44, NKp46, KIRs in humans, Ly49 receptors, in mice have been identified (5). The combination of the activation receptors can synergistically mediate natural cytotoxicity (6). NKG2D, a type II transmembrane-anchored C-type lectin-like activating receptor, is a well-characterized activation receptor expressed by NK cells, NKT cells, activated CD8^+^ T cells, subsets of γδ^+^ T cells, and innate-like T cells, which are TCR^+^ NK1.1^+^ CD49a^high^ CD103^+^ tissue-resident T lymphocytes with innate cytolytic activities, transcriptionally related to ILC1 (7–11). NKG2D can recognize multiple ligands (NKG2D-ligands), which are homologous to MHC class I molecules; MHC class I chain-related proteins A (MICA), MICB, UL16 binding protein 1 (ULBP1)–ULBP6 in human; retinoic acid early inducible 1 (RAE-1) (isoforms α–ε), H60 (isoforms a–c), and murine UL16-binding protein like transcript 1 (MULT1) in mouse (12). Interaction of these ligands with NKG2D results in NK cell cytotoxicity *via* signal transducing adapter molecule DAP10 in human and both DAP10 and DAP12 in mouse (10). Surface expression of NKG2D-ligands on healthy cells is tightly restricted by regulation at transcriptional and posttranscriptional levels, to ensure that healthy cells are not recognized by the innate immune system. The mechanisms involved in NKG2D-ligand expression regulation have been studied extensively [reviewed in Ref. (12, 13)].

Emerging evidence shows that intracellular stress can also induce the NKG2D-ligand expression. In this review, we summarize the mechanisms of NKG2D-ligand regulation. We focus specifically on recent advances in our understanding of how endoplasmic reticulum (ER) stress leads to NKG2D-ligand surface expression, and eventually group 1 innate lymphoid cells (ILCs)-mediated inflammation, particularly inflammatory bowel diseases, which are associated with several ER stress-related genes. In addition, we discuss the mechanisms by which NKG2D-L are suppressed on the other hand and specifically through carcinoembryonic antigen (CEA)-related cell adhesion molecule 1 (CEACAM1).

## Regulation of NKG2D-Ligands

### Regulation of NKG2D-Ligands by Cellular Stress and ER Stress

As NKG2D-ligand expression signals the immune system to recognize infected or transformed cells, a variety of stress pathways have been demonstrated to regulate NKG2D-ligand expression *via* different mechanisms (Table 1). Oxidative stress leads to accumulation of H_2_O_2_, which induces NKG2D-ligand including MICA/B and ULBP1–4 *via* activation of the mitogen-activated protein kinases pathway (14, 15). In contrast, heat shock can transcriptionally regulate MICA/B, as the promoter regions of the MIC genes have heat shock elements that can be recognized by heat shock factor 1 (HSF1) (15–17). Knockdown of HSF1 has been shown to suppress MICB, but not MICA, membrane expression leading to a reduction in NK cell-mediated cytotoxicity (18). In mice, heat shock induces MULT1 protein expression in fibroblasts and transformed cells by altering protein stability (19). One of the mechanisms associated with regulation of MULT1 surface expression by heat shock could be the membrane-associated RING-CH (MARCH) family of E3 ubiquitin ligase. While MULT1 is post-transcriptionally regulated by ubiquitin-dependent degradation by the MARCH family in unstressed cells, MULT1 ubiquitination and degradation are reduced in response to heat shock stress (19, 20).

**Table 1:** Natural-killer group 2 member D (NKG2D)-ligands regulation by cellular stress.

Type of cellular stress	Regulation mechanisms	Mouse NKG2D-ligands	Human-NKG2D ligands	Reference
Oxidative stress	MAPK pathway		MICA/B, ULBP1-4	(14, 15)
Heat shock	Transcriptional (HSF1)		MICA/B	(15–20)
	Ubiquitin-dependent degradation (E3 ubiquitin ligase)	MULT1		
Endoplasmic reticulum stress	Transcriptional (ATF4, CHOP)	MULT1	ULBPs	(25, 26)

*ATF4, activating factor 4; CHOP, CCAAT/enhancer-binding protein (C/EBP) homologous protein; HSF1, heat shock factor 1; MAPK, mitogen-activated protein kinases; MICA/B, MHC class I chain-related proteins A/B; MULT1, murine UL16-binding protein like transcript 1; ULBP1, UL16 binding protein 1*.

Until recently, little was known about how cellular stress that leads to disturbances in proteostasis, and eventually ER stress, interact with regulation of NKG2D-ligand expression. ER stress is caused by the accumulation of unfolded and misfolded proteins in the ER arising from either primary (genetic) or secondary (environmental) factors (21). Highly secretory cells are highly susceptible to ER stress. These include Ig-producing plasma cells, insulin-secreting β-cells in the pancreas and intestinal epithelial cells, in particular Paneth cells and goblet cells (22). ER stress leads to the accumulation of unfolded or misfolded proteins within the ER lumen, which triggers three ER stress sensors to induce the so-called unfolded protein response (UPR). These include inositol-requiring transmembrane kinase-endonuclease 1 (IRE1), protein kinase RNA-like ER kinase (PERK), and activated transcription factor 6 (22). The main goal of UPR activation is to restore proteostasis and enhance the secretory capacity of the ER. In the IRE1 arm of the UPR, phosphorylated IRE1 possesses endoribonuclease activity that excises a 26-nucleotide sequence of *X-box binding protein 1* (*Xbp1*) mRNA resulting in a frame shift and generation of a transcriptionally active isoform that functions as a transactivator of UPR target genes.

In the PERK arm, translation is suppressed by phosphorylation of elongation initiation factor 2α (eIF2α) to allow the cell to temporarily cope with excessive ER stress. Paradoxically, translation of some proteins, such as activating factor 4 (ATF4) is favored. Initially, ATF4 can induce several protective cellular pathways, among others autophagy (see below). However, if ER stress is excessive or prolonged, ATF4 induces apoptosis-related transcription factors, such as CCAAT/enhancer-binding protein (C/EBP) homologous protein (CHOP) (23).

Besides cell death, ER stress elicits inflammatory responses, and hence it was hypothesized that UPR-related proteins can induce surface expression of NKG2D-ligands. Surprisingly, Xbp1 knockdown in the mouse immortalized small intestinal epithelial cell line MODE-K (24) (*shXbp1* MODE-K by a short hairpin Xbp1 lentiviral vector), which causes ER stress (25), was shown to induce very strong induction of NKG2D-ligand MULT1 (both on mRNA level and surface protein expression), whereas inflammatory signals induced after stimulation with a variety of TLR ligands did not (25). Even more interesting was the fact that it appeared to be specific for MULT1, as both RAE-1 and H60 were not strongly induced. In contrast, expression of MHC class I, which is recognized by NK cell inhibitory receptors, was not affected by *Xbp1* knockdown *in vitro* and knockout *in vivo*. The effect was not specific for *Xbp1* deletion, as ER stress induction by administration of thapsigargin similarly induced strong upregulation of MULT1 surface expression. In addition, similar induction of ULBPs (the human ortholog of MULT1) was observed in a variety of human cell lines, including intestinal, gastric, esophageal, and hepatic cancer cell lines.

**Figure 1:**
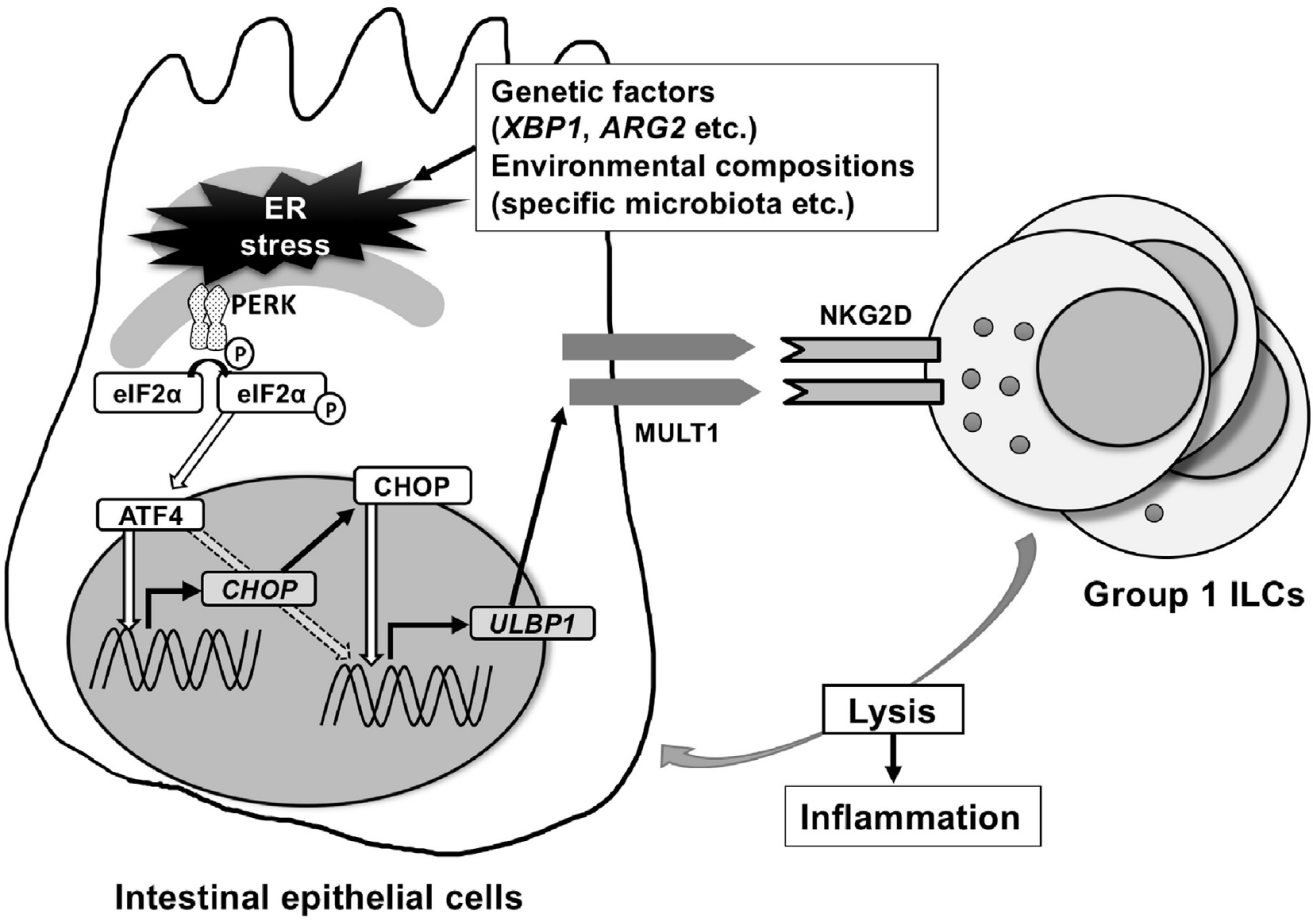
Endoplasmic reticulum (ER) stress-inducing murine UL16-binding protein like transcript 1 (MULT1) in mouse. MULT1 [encoded by UL16 binding protein 1 (*Ulbp1*)], but not other natural-killer group 2 member D (NKG2D)-ligands, mRNA, and surface expression are upregulated on ER-stressed mouse small intestinal epithelial cells. This occurs through activating factor 4 (ATF4) and/or C/EBP homologous protein (CHOP) presumably downstream of the protein kinase RNA-like ER kinase pathway. CHOP as a transcription factor binds directly to the promoter area of *Ulbp1*, which upon expression subsequently activates NKG2D-expressing intraepithelial group 1 innate lymphoid cells (ILCs) [natural killer (NK) cells and ILC1] that accumulate within the intestinal epithelium and promote small intestinal inflammation.

Intriguingly, ER stress protein ATF4 was found to be important in NKG2D-ligand upregulation using a completely different approach in a human cancer cell line HAP1 (26). This cancer cell line constitutively expresses ULBP1 and after treatment with a retroviral promoter trap vector, which randomly knocks out genes, the cell lines that had significant downregulation of ULBP1 surface expression were screened for gene enrichment. This screen revealed that ATF4 is important for the induction of ULBP1, which was confirmed by demonstrating that knockdown of ATF4 strongly decreased ULBP1 transcription. In addition, ATF4 was shown to have direct ULBP1 promotor binding sites and directly transactivates the ULBP1 promoter (26). In contrast to this study in human cancer cell lines, we have identified CHOP as a transcription factor that binds the promoter of the mouse ortholog of ULBP1, MULT1, using chromatin immunoprecipitation and luciferase assays. CHOP is downstream of PERK-ATF4, but can also be induced by other ER stress-associated pathway elements (27). Interestingly, MODE-K cells with silenced CHOP using *shRNA*, or primary intestinal epithelial cells from *Chop*^−/−^ mice examined *ex vivo* show downregulation of ER stress-dependent induction of MULT1 on the surface of intestinal epithelial cells (Figure 1).

Together, these two recent studies indicate that the PERK–ATF4–CHOP pathway of the UPR is a highly conserved ER-stress-specific mechanism of regulation of NKG2D-ligands. Interestingly, ER stress is a common pathophysiological phenomenon in the two disease processes that have been mostly linked to NKG2D-ligand expression, namely cancer, discussed below, and (viral) infection. Viral infection can strongly induce NKG2D-ligands. For example, human immunodeficiency virus type 1 (HIV-1) induces DNA stress/damage checkpoint arrest initiated by the DNA damage-sensing protein kinase (ataxia telangiectasia-mutated and Rad3-related: ATR) (28), resulting in upregulation of NKG2D-ligands (29, 30). Recognition of viral products, such as double-stranded (dsRNA), by retinoic acid-inducible gene I and melanoma differentiation-associated gene 5, both cytoplasmic pattern recognition receptors, also upregulate MICA and ULBP2 expression (31). Since viral replication requires the host ER for the production of their structural and non-structural proteins, viral infection has been strongly associated with activation of the UPR as well, and further research is required to investigate how the UPR in viral infection affects NKG2D-ligand expression.

### Consequences of ER Stress-Induced Regulation of NKG2D-Ligands in the Gastrointestinal Tract

In genome-wide association studies, MICs gene have been associated with susceptibility to a variety of autoimmune diseases, including systemic type 1 diabetes, rheumatoid arthritis, and lupus erythematosus (32–34). Consequently, NKG2D/NKG2D ligand interactions have been hypothesized to be involved in their pathogenesis (35–38).

In the intestine, NKG2D/NKG2D-ligand interactions have mainly been studied in celiac disease. Celiac disease is a gluten-driven innate and acquired immune cell-mediated enteropathy characterized by findings of intraepithelial lymphocytosis, crypt hyperplasia, and villous atrophy, and a positive response to a gluten free-diet (39). The fractions of NKG2D^+^ NK cells and NKT cells among the intraepithelial mononuclear cells in active celiac disease are significantly increased as compared to inactive celiac disease or control subjects (40). Increased MICA expression on the intestinal epithelium in active celiac disease *via* a pathway involving IL-15 induction by gliadin triggers activation of intraepithelial T cells, allowing for the killing of intestinal epithelial cells (41). Details of the MICA/B expression pattern showed that the expression was observed not only on epithelial cells but also in the cytoplasm of intraepithelial T lymphocytes in patients with active celiac disease, suggesting extensive stress conditions are present in active celiac disease (42). Interestingly, some studies have suggested that ER stress could be involved in NKG2D-ligand expression regulation in celiac disease (42).

In Crohn’s disease, several studies have also suggested involvement of the NKG2D/NKG2D-ligand pathways in the pathogenesis of this disorder (43–45). Specifically, MICA and MICB expression has been demonstrated to be increased on intestinal epithelial cells in patients with inflammatory bowel disease (46, 47). Moreover, a subset of CD4^+^ T cells expressing NKG2D was increased in patients with Crohn’s disease and functionally active through MICA–NKG2D interactions, leading to interferon-γ production. In pediatric Crohn’s disease and ulcerative colitis, ULBP1 and ULBP2 is upregulated on infiltrating immune cells in active inflammatory lesions (48). Interestingly, a recent clinical trial revealed efficacy of a blocking anti-NKG2D IgG4 antibody in patients with active Crohn’s disease (49), suggesting that NKG2D/NKG2D-ligand interactions are of importance in the pathogenesis of Crohn’s disease.

As ER stress has also been linked to the pathogenesis of Crohn’s disease (50), and as we demonstrated that ER stress regulates NKG2D-ligands on intestinal epithelial cells, we subsequently investigated the functional consequences of epithelial ER stress-induced NKG2D-ligand expression and its role in the development of spontaneous enteritis in a mouse model of this condition. We demonstrated that increased MULT1 expression on intestinal epithelial cells of *Xbp1*^Δ^*^IEC^* mice was accompanied by increased quantities of NKG2D-expressing intraepithelial group 1 ILCs (NK cells and ILC1) (25). The group 1 ILCs within the epithelium also exhibited evidence of increased activation as demonstrated by increased surface expression of NKG2D and CD25. Indeed, IELs from *Xbp1*^Δ^*^IEC^* mice exhibited increased cytotoxicity in comparison to the activity observed with IELs from wild-type mice, suggesting an involvement in inflammation, which was supported by amelioration of inflammation upon NKG2D blockade. Furthermore, ER stress-induced intestinal inflammation due to *Xbp1* deletion in the intestinal epithelium was uniquely comprised of a significant component of innate immune activation as concomitant loss of the adaptive immune system in *Rag1^−/−^Xbp1*^Δ^*^IEC^* double mutant mice did not affect the severity or kinetics of the inflammation. However, depletion of NK cells significantly diminished inflammation that emerged from tamoxifen-driven, and thus temporally controlled induction of ER stress during adult life in *Xbp1^T-^*^Δ^*^IEC^* mice. Similarly, spontaneous enteritis was reversed by NKG2D blockade in *Xbp1^T-^*^Δ^*^IEC^* mice, in line with what has been shown for mouse colitis models (51, 52). Thus, our study demonstrates that ER stress in the small intestine leads to spontaneous enteritis that depends significantly on the presence of an innate immune system. Further, the development of ER-stress-mediated inflammation in this context involves the CHOP-dependent induction of NKG2D-ligand MULT1 and its recognition by NKG2D on group 1 ILC, which are increased in the intestinal epithelium. Future studies are required to investigate how ER stress is linked to NKG2D-ligand expression in the human gut, particularly in the setting of complex diseases including inflammatory bowel disease. Methods to resolve pathologic levels of ER stress could, therefore, be a potential target for therapies in this still uncurable disease.

### Regulation of NKG2D-Ligand Expression in Cancer

NKG2D-ligand expression has been extensively studied in the setting of cancer. The expression of NKG2D-ligands in cancer can be hypothesized to serve in activating the immune system for elimination of the excessively proliferating cancer cells. E2F transcription factors, for example, regulate cell proliferation but at the same time can induce specific NKG2D-ligands such as RAE-1, but not MULT1 and H60 in mouse fibroblasts by directly binding the promoter region of *Raet1* genes (53).

DNA damage responses to genotoxic stress coordinates activation of transcription, cell cycle control, apoptosis, and DNA repair processes mediated by a number of protein kinases, including ATM (ataxia telangiectasia mutated) and ATR (ATM and Rad3-related) protein kinases (54). In both mouse and human non-tumor cells, genotoxic stress and inhibition of DNA replication *via* the DNA damage pathway through ATM or ATR protein kinases, leads to increased surface expression of NKG2D-ligands (55). In tumor cell lines, constitutive ligand expression on the cell surface is suppressed by pharmacological or genetic inhibition of ATR, ATM, or Chk1 (55).

p53, induced in response to DNA damage, can also regulate human ULBP1 and ULBP2 by binding to p53-responsive elements in the promoter area of *ULBP1* and *ULBP2* gene (56–58). However, several microRNAs that are induced by p53 (miR-34a and miR-34c) have been shown to suppress ULBP2 expression by directly binding to the 3′-UTR of *ULBP1* mRNA (59).

Other mechanisms of NKG2D-ligand induction in response to DNA damage response include the stimulator of interferon genes (STING)-dependent DNA sensor pathway (60). The accumulation of cytosolic DNA by DNA damage response activates STING-dependent DNA sensors, leading to the activation of TANK binding kinase 1 and interferon regulatory factor 3, which in turn are associated with RAE1 expression (60).

### Inhibition of NKG2D-Ligand Expression on Tumor Cells by CEACAM1

As NKG2D-ligand expression on tumor cells is critical for the recognition and clearance of tumors, tumor cells have evolved by developing several mechanisms to escape from immune surveillance. Several studies have demonstrated that soluble forms of NKG2D-ligands that were derived from cancer cells by either proteolytic shedding (61–63), alternative splicing (64), or exosome secretion (65) can impair NKG2D-mediated cytotoxicity by negatively regulating NKG2D expression or recognition (66). In addition, metastasis-associated microRNA miR-10b (also known as metastamir), which promotes tumor invasion and metastasis by targeting multiple genes, downregulates MICB expression by binding directly to the 3′-UTR of *MICB* (67).

**Figure 2:**
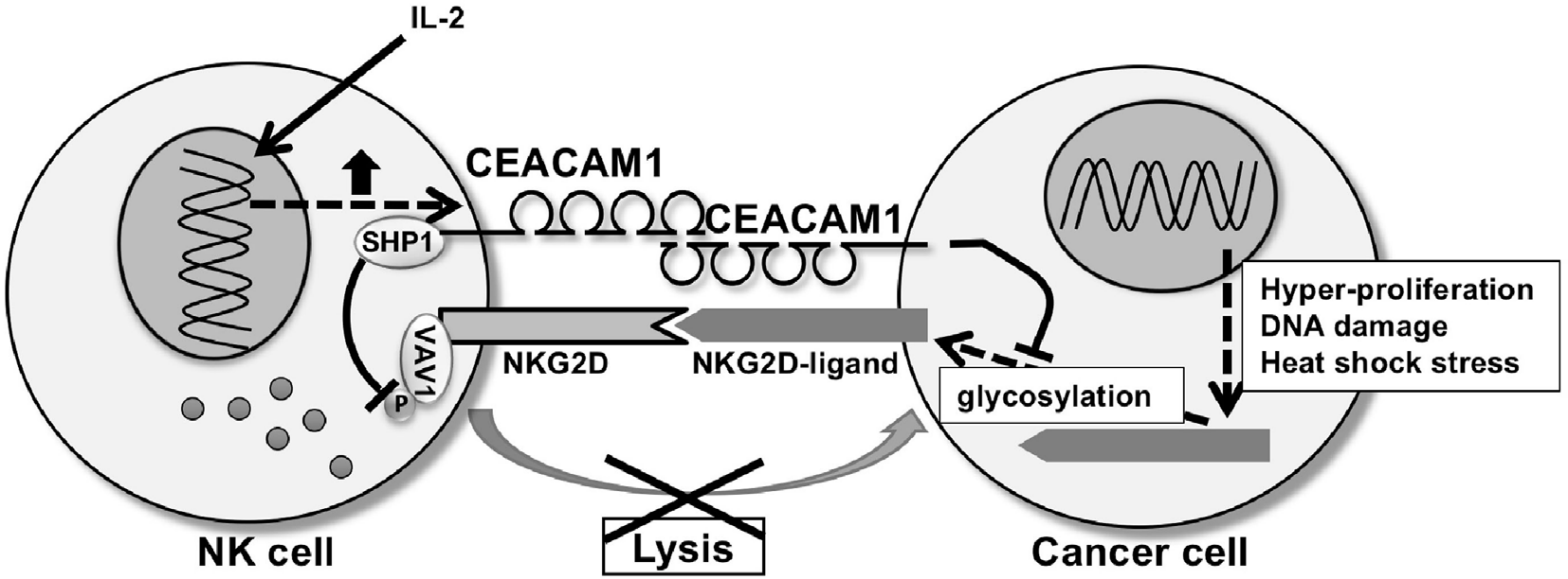
CEA-related cell adhesion molecule 1 (CEACAM1) regulating natural-killer group 2 member D (NKG2D)-ligands expression and NKG2D function. CEACAM1 regulates glycosylation of NKG2D-ligands, resulting in downregulation of the NKG2D-ligand expression on the cell surface of the tumor cell. CEACAM1 induced by interleukin-2 inhibits NKG2D-mediated cytotoxic function through recruitment of Src homology phosphatase 1 (SHP1) to phosphorylated CEACAM1 which leads to dephosphorylation of Vav1 on the natural killer (NK) cell. CEACAM1 on the NK cell and tumor cell interact homophilically through the N-domain of CEACAM1.

CEACAM1, a member of the CEA family of Ig like transmembrane glycoproteins (68), is involved in the negative regulation of NKG2D-ligands in cancer (69–71). CEACAM1 is expressed in mouse and humans and is characterized by numerous transmembrane isoforms that derived from alternative splicing mechanisms [reviewed in Ref. (68, 72)]. This mechanism generates CEACAM1 variants that share a membrane distal IgV-like domain (N-domain), which functions in homophilic or heterophilic interactions, that is coupled to variable numbers of IgC2 domains and linked to either a long (L) or short cytoplasmic domain. NK cells and T cells predominantly express CEACAM1-L isoforms that contain two immunoreceptor tyrosine-based inhibitory motifs in their cytoplasmic tail which serves to recruit Src homology phosphatase 1 (SHP1) and SHP2 after phosphorylation by Src-related kinases (68, 73). Ligation of CEACAM1-L isoforms on NK cells by CEACAM1 on tumor cells suppresses NK cytolytic function as CEACAM1 on the NK cells negatively regulates NKG2D signaling (74). Specifically, recruitment of SHP1 by CEACAM1 leads to dephosphorylation of the guanine nucleotide exchange factor Vav1, one of the most proximal elements associated with NKG2D-mediated cytolytic signaling (75). At the same time, CEACAM1 expression on the tumor cells has been shown to regulate NKG2D-ligand expression. Specifically, silencing of CEACAM1 in mouse and human tumor cells upregulates expression of NKG2D-ligands on the cell surface of the tumor cell and makes them more highly susceptible to NK cell-mediated cytotoxicity (Figure 2) (76). Silencing CEACAM1 did not alter the transcriptional levels of NKG2D-ligands. Indeed, cell surface expression of RAE-1 was increased in CEACAM-1 silenced cells, whereas intracellular RAE-1 protein was decreased in comparison to non-silenced cells. Furthermore, RAE-1 on the cell surface of CEACAM1 silenced cells possessed an increased quantity of carbohydrate side-chain modifications in comparison to CEACAM1 non-silenced cells, in which RAE-1 accumulates intracellularly as an incompletely glycosylated protein (76). Thus, CEACAM1 can regulate glycosylation of NKG2D-ligands resulting in the NKG2D-ligands retention in an intracellular compartment.

## Conclusion

NKG2D/NKG2D-ligands interactions play a critical role in the immune surveillance of sick cells, such as those that are infected or have undergone neoplastic transformation. In such contexts, the expression and function of NKG2D and NKG2D-ligands are critical for removal of the altered cells and resolution of the condition. However, in certain circumstances cancer and infected cells have developed the ability to avoid immune surveillance that is mediated by NKG2D/NKG2D-ligands which allows progression of the infection or tumor. Our recent studies indicate that CEACAM1 plays an important role in NKG2D/NKG2D-ligand function and expression, respectively, suggesting that manipulation of this pathway may be beneficial in such disorders. On the other hand, NKG2D/NKG2D-ligand expression and function may inappropriately be associated with distressed organs or cells in autoimmune disease, suggesting that their blockade would be an important means of resolving inflammation consistent with their current evaluation in clinical trials in inflammatory bowel disease (49). Our recent studies thus suggest that enhancement of CEACAM1 function might be beneficial in such disorders by enhancing inhibition of NK and ILC1-mediated activation due to NKG2D/NKG2D ligand interactions. This is consistent with previous studies that application of CEACAM1 ligands to mouse models of colitis can suppress inflammation (77). This is particularly interesting as a key feature of inflammatory bowel disease which is the common induction of ER stress within the intestinal epithelium, which can serve as nidus for development of inflammation (22, 50). As such, the recent evidence that ER stress is linked to the upregulation of specific NKG2D-ligand and activation of intraepithelial NK cells and ILC1, together imply that blockade of NKG2D and NKG2D ligand interactions may have wide benefit in inflammatory bowel disease and potentially other autoimmune conditions.

## Author Contributions

All authors wrote and edited the manuscript and gave final approval of the manuscript.

## Conflict of Interest Statement

RB is a consultant to Syntalogic Pharmaceuticals which is developing therapies that target CEACAM1. The other authors have no conflict of interest.
